# Conference to Form a London Region

**Published:** 1920-12-11

**Authors:** 


### THE BRITISH HOSPITALS' ASSOCIATION.

### Conference to Form a London Region.

A well-attended meeting of members of the Association was held in the Barnes Hall, Royal Society of Medicine, on December 2, to confer on the desirability of forming a Regional Committee for London.

The chair was occupied by the Hon. Sir Arthur Stanley, who was supported by Viscount Knutsford, Lord Goschen, Colonel Mildmay, Sir Napier Burnett, and others.

The Secretary (Mr. J. C. Buchanan) read the following letter, which had been received from the Secretary to Charing Cross Hospital:

"In reply to your circular letter of the 23rd inst., I am instructed to say that, while the authorities of this hospital are quite in sympathy with your Association, we are working here on such entirely different lines to nearly every other hospital in London, that we could not agree to be included in the conclusions drawn on behalf of the London hospitals generally at this time, especially when legislation of a most controversial character is threatened, and it is most desirable, in our opinion, that a public inquiry should be held at the House of Commons, where the views of each individual hospital should be heard. In other words, we have decided, but in no truculent spirit, to preserve our independent attitude; and we must ask you, therefore, in any representation of the report made by your Association, to state clearly that this hospital is not included."

### Regional Committees.

The Chairman: I take it that if we can do anything which is of real use, the Charing Cross Hospital will not mind taking advantage of it. (Laughter.)

Perhaps you will allow me to say a few words about the reason of this meeting. For a good many years past the British Hospitals' Association has existed. Before the war, in what we look back upon as the palmy days of voluntary hospitals, there was not much for this Association to do. During the war, of course, there was practically nothing for it to do. But when the end of the war came, when we found the voluntary hospitals of the country in a very serious position, it became very clear that one quite necessary thing was an Association that could gather the opinions of the voluntary hospitals generally, and express them in a way to command the respect of the Government and of Government Departments. It was obviously not to be expected that the voluntary hospitals throughout the country would send their representatives up frequently to London, and we therefore decided that the best way to reach the general opinion of the voluntary hospitals was to divide the country into regional areas, to get strong Committees in all those areas, and link them up with a Committee here at headquarters. With the Council of the Association the formation of these regional areas has been going on during the past twelve months. We have, for convenience, followed, as far as possible, the areas laid down by the Ministry of Pensions, which seemed to us a convenient division. Very satisfactory Regional Committees have already been formed in most parts, and we are in process of getting nearly the whole country covered in this way. We shall have these committees in different parts of the country, which will be in close touch with us at headquarters. We place the committees in a central town, so that they will be of easy access from the whole of the district which they will serve. In that way we feel we shall be in close connection with the voluntary hospitals throughout the country. Scotland has also formed a Regional Committee, and will be in connection with us.

Obviously, a very important Regional Committee to form is that of London. We have dragged rather behind in forming this Committee, and that has been for various reasons. In the meantime the Hospitals Association Council, on its own initiative, nominated a Committee to deal with matters as they affected the voluntary hospitals of London. What is known as the Hambleden Committee has been sitting, under the chairmanship of Lord Hambleden, during the last few months, and we owe to that Committee a very great debt of gratitude for having shown, under his guidance, the real importance it must have for the voluntary hospitals of London. It is necessary to have a scheme, and the proposal will be to consider the desirability of forming this Committee. But if you settle that a Committee of this kind is to be formed, it is as well to have some basis for discussion. I have ventured to put into your hands a suggestion as to the way that Committee might be formed. I will not say, at the moment, more than this: that I think it is desirable, in a Committee of this kind, not to appoint individual persons, but to appoint hospitals, and groups of hospitals, and get them to send a representative. The reason of that is that it is necessary for us to get the best opinion on any particular subject which comes uppermost. It is not likely that one individual who might be appointed a representative by a hospital would be the best man that they could have on finance and on, say, nursing too. Therefore we want to leave it free to different hospitals, or groups of hospitals, to appoint, for a particular meeting, that representative who they think will best voice their opinions on the subject which will be discussed at that particular meeting of that Committee. I will call upon Sir Edward Penton, of Middlesex Hospital.

### The Question of London.

Sir Edward Penton moved the resolution, which was: "That the Association shall form a Regional Committee of the British Hospitals Association for London." He said:

In my view we should be very careful that this is in no way an autocratic Committee. There is one very distinct school of thought in this country, which is held by people who, in other things, are absolutely at two opposite poles. There is a certain amount of beaurocratic element which believes it can run every institution in this country by bringing it under the Government ægis. And there is the extreme thought, on the other hand, that is probably known as the extreme Labour thought, which believes every institution should be run by the State. In between those two schools of thought the large mass of the population would like, in some may or other, to maintain its old systems and traditions, properly adapted to modern circumstances. We must realise there must be changes in method; we must realise we cannot all remain stationary. And yet, realising that, there is no necessity to throw the whole cargo overboard and immediately swim for the ship of State. Probably Members of Parliament among us will tell us the ship of State would not be able to carry us. If those views are to be clearly expressed, surely there must be some central authority or central body which is able to express clearly, properly, and authoritatively, the general considered views of the great majority of the people engaged in the management of the voluntary hospitals. It is not a new scheme by any means. Nearly all trades are doing it now. The Federation of British Industries, a body which federates trades, asked Mr. Lloyd George to dinner the night before last to discover what his views on the future of British trade were. Nearly every trade has its Chamber of Commerce, which discusses, and rightly discusses, problems common to all people engaged in trade, not in any way interfering with the individuality or the competition of the various units. I cordially propose the resolution requiring the formation of this Committee, and I trust members will agree to it, I hope, on the lines laid down.

Lord Goschen seconded the resolution.

Sir Samuel Scott asked as to what body this Regional Committee must report.

The Chairman replied that it would report to the Council of the Association, and he hoped that next year that Council would consist of delegates appointed by the Regional Committees in all parts of the country. Answering a further

**British Hospitals' Association**—(*continued*).

question, he said it would really be an acting Committee, a working Committee of the Council. It would be impossible every time a question needed discussing to call a meeting of representatives of all the London hospitals, therefore they wished to form a Committee which could be got together more easily.

The resolution was carried without dissent.

The Chairman: If it is your pleasure we will now proceed to a discussion on how that Committee is to be formed. The proposal before you is as follows:

| | Beds. | | | Delegates |
|---|---|---|---|---|
| The 12 large hospitals with medical schools with a total of ... ... ... ... ... | 5,431 | | nominate | 12 |
| The 8 larger general hospitals without medical schools with a total of ... ... ... | 1,439 | | ,, | 4 |
| The 13 smaller general hospitals without medical schools with a total of ... ... | 922 | | ,, | 3 |
| | | 7,792 | | —19 |
| The consumptive hospitals with a total of ... | 508 | | | 1 |
| The women's hospital with a total of ... ... | 296 | | | 1 |
| The children's hospital with a total of ... | 669 | | | 1 |
| The eye hospitals with a total of ... ... | 258 | | | 1 |
| The nerve hospitals with a total of ... ... | 354 | | | 1 |
| The cottage hospitals with a total of ... ... | 413 | | | 1 |
| The lying in hospitals with a total of ... ... | 375 | | | 1 |
| The 34 non-classified special hospitals ... | 2,216 | | | 6 |
| | | 5,089 | | —13 |
| Grand total ... | | 12.881 | | 32 |

One representative for each of these large classes does not seem very much, but, on the other hand, I think it may have this advantage: that it will, I hope, get each group of hospitals in the habit of meeting to discuss questions which particularly affect their group of hospitals. That would be a great advance. The total would be a Committee of thirty-two, which is, I think, a very good size for a Committee, as it would be quite manageable. In this scheme we have endeavoured to see that all interests are represented. I shall be very grateful for any criticisms. It is based, roughly, on the idea of King Edward's Fund.

After some discussion the motion was carried by a large majority.

Sir Samuel Scott moved, and Lord Govell seconded, an addition to the resolution providing for the co-option of any gentleman to represent a hospital on a particular subject, and this, with the original resolution, was agreed to.

Colonel Mildmay, M.P., proposed: "That this Conference of Chairmen and Representatives of the Governing Bodies of Voluntary Hospitals in London welcomes the appointment by the Minister of Health of a small committee 'to inquire into and report upon the financial position of the voluntary hospitals throughout the country, and to make recommendations.'" In commending it to the meeting he said: My knowledge of the management of hospitals is not so great as that of many I see before me, not so great as Lord Knutsford's, for example; but I have taken much interest in hospital management in a particular institution. The reason I have come before you is that in the House of Commons we have had this Ministry of Health Bill before us. It was about to be debated in a Standing Committee, but many Members of Parliament were absolutely ignorant with regard to the conditions in hospitals, and as to what was required. Some of them came and said, "We are anxious to do our duty under these circumstances to hospitals, but we are ignorant." The only chance to get what they wanted was to get together a meeting of members of the House of Lords and the House of Commons who were interested in the management of hospitals and bring them into contact with those other members. We did so, and that meeting was very successful. When a member of Parliament asked what is the thing to do in the financial condition in which many hospitals are, it was difficult to answer, because the circumstances of many hospitals in London vary so much with regard to endowment and the wealth of the district they serve, and in many other respects. We wanted the matter tackled at once, and we impressed upon Dr. Addison that the committee must be appointed forthwith. Dr. Addison, I am glad to say, met us in every way: Sir Arthur Stanley and I went to see him. He gave us an hour and a-half of his time, and we talked over the whole matter thoroughly, and he accepted all our views. As to the composition of the committee, we felt so strongly it was desirable to have a committee upon which representatives of individual hospitals should not have a place, because the thoughts of all go back to our own hospital. We wanted a good man as Chairman, one of independent thought, who had some knowledge of finance, and a very few independent men besides him. Dr. Addison met us in every way, and we have cause to be very grateful to him. During a long experience in the House of Commons I have found that in approaching Government authorities the thing is not to go to them in an aggressive attitude, but to take it for granted that they will conceive that everything you say must be right, and they will agree with you. That Sir Arthur and I did, and Dr. Addison agreed with us; and I look forward to real good work by this Committee which is about to be appointed, because it is most essential that the valuable opinions may be obtained from those who manage hospitals, and should be co-ordinated in such a form as to be available for those who frame legislation. That will be in the future. I need say no more, I think, in asking you to give hearty support to this resolution.

Lord Knutsford: In seconding this, I shall be very short, because my feelings get the better of me. I never before heard that Members of Parliament were ignorant. On this occasion they managed to disguise their ignorance when they got the Minister of Health to help them. If I do not make a long speech it is not because I am afraid of boring you. If this is not a funeral breakfast, it is a meeting at the bedside of a sinking relative. ("No.") But because we feel inclined to weep is no reason why the patient should not get better, and even rise again. And I hope this committee which Dr. Addison will appoint will consist of men not necessarily connected with any hospital, but men of independent judgment and some experience, not necessarily in hospital work. I am sure the Minister will do his best to appoint men of that calibre. And it is important that we should have acting for that committee a common auditor for all, because often the accounts are presented in so many different ways, and we want to get them all down to a common denominator. And I hope it will be impressed upon this committee that they must report quickly, because if their report is to be simply on the financial position of the hospitals they could get that information in a week. But I hope they may go further, and point out not only the financial position of the hospitals and how that might be remedied, but they must remember that next March is the danger moment for all hospitals, because the quarterly accounts come in in March, and that will be the dangerous time, the crisis for the patient. Therefore we hope they will report as quickly and as strongly as possible.

The Chairman: I do not agree with Lord Knutsford that this is a funeral meeting; I think it is in the nature of a *conseil de famille*—*i.e.*, someone being called in to look after one refractory member in the interests of the member himself. (Laughter.) I would like to add to what Col. Mildmay said. Dr. Addison met us in the most frank and friendly way, and I am certain his genuine wish is to do what is best for the voluntary hospitals. And I think this Committee which he is setting up will be of infinite advantage to the Minister himself and to the voluntary hospitals. I have, therefore, very great pleasure in putting this motion. I think that with it we could convey our appreciation of the way in which Col. Mildmay and others have been met by Dr. Addison.

Carried.

The Chairman: It will be for the Committee which we have set up to-day to bring before the Committee appointed by the Minister questions concerning the voluntary hospitals, and I would have liked the meeting to have gone into them to-day, but it is too late. Perhaps, however, Sir Napier Burnett will say a brief word.

Sir Napier Burnett: I have been present, along with your Chairman, at the opening of two or three Centres, when he has acted as the chief accoucheur at the birth of these young bodies. They are full of energy. Another starts at Leeds, and I hope to be present on Wednesday

British Hospitals' Association—(*continued*).

next at that new centre of this Association. I am sure we are tremendously relieved that the Ministry has set up this Committee with the object of getting the facts and then proceeding to legislation. I think that is the right order of events. And one of the fine things of that Committee to those of us who are more in touch with finance will be that the voluntary system will require some supplementary aid. The second point is, what is going to be the source and the nature of that aid? If there had been a resolution coming from the voluntary bodies represented here as to whether they were in favour of the aid coming from the rates or from the State, it would have been helpful. Dr. Addison has appealed for help from the British Hospitals Association; he has wanted guidance from such a body as this, but he has not yet got it; and I thought that to-day it would have been well to have passed such a resolution. I hope another step will be the establishment of a Central Hospitals Board for this country; it is very much needed. If the money should come from the State I would indicate a possible source of that money. If you are interested in the matter and will get the publication issued by the Treasury a few months ago you will find there is a very interesting statement which deals with the amounts of money which have been expended on social services. It is brought out by Mr. Stanley Baldwin, a representative of the Treasury on the Front Bench. Under the National Insurance Health Act, 1911, the total expenditures were £17,330,000—that is, £13,700,000 for benefits, and the administration expenses £3,630,000—an interesting figure. But the receipts came to £24,400,000, so there was a surplus from that Act, in one year, of £9,100,000. Lord Knutsford asks where is that money. I want to know that too. I understand there is a big sum of money—fifty odd millions—lying somewhere, and I suggest, in all seriousness, that this gets at the crux of the matter; your difficulties have come upon you since the National Insurance Act came into operation. It is because of that Act you are flooded with patients; you are doing the work of these hospitals for this National Insurance Act, but no hospital benefit was included in it. Why are those millions lying in reserve? I am told no money from this fund can be obtained until there is an amendment in the Act to permit the hospital benefit to come under it. And if there were a Central Hospitals Board set up it might get the money distributed to the hospitals for their needs. I put that forward as an instructive suggestion for this Committee, which we are indebted to Dr. Addison for having agreed to have set up.

The Chairman: We shall have, as soon as possible, a meeting of the Committee which you have set up to-day, and we shall bring forward suggestions as to the formation of a Central Hospitals Board. We have a very good analogy in the case of the formation of the University Grants Board.

The Unemployment Insurance Act.

The Chairman said he had received a letter from the Ministry of Labour on the subject of the Unemployment Insurance Act, because it was giving them trouble. This intimated that the Minister would give his decision concerning the position of probationery nurses, staff nurses, and sisters, and send a communication embodying it. Also in regard to hospital porters.

A vote of thanks to the Chairman concluded the meeting.

